# Effect of Aromatic Chain Extenders on Polyurea and Polyurethane Coatings Designed for Defense Applications

**DOI:** 10.3390/polym15030756

**Published:** 2023-02-02

**Authors:** Gabriela Toader, Andreea Elena Moldovan, Aurel Diacon, Florin Marian Dirloman, Edina Rusen, Alice Podaru, Traian Rotariu, Raluca Elena Ginghina, Oana Elisabeta Hoza

**Affiliations:** 1 Military Technical Academy “Ferdinand I”, 39–49 George Cosbuc Boulevard, 050141 Bucharest, Romania; 2 Faculty of Chemical Engineering and Biotechnologies, University of Bucharest, 1–7 Gh. Polizu Street, 011061 Bucharest, RomaniaRomania; 3 Research and Innovation Center for CBRN Defense and Ecology, 225 Oltenitei Ave., 041327 Bucharest, Romania; 4 Faculty of Material Science and Engineering, University of Bucharest, 313 Splaiul Independentei, District 6, 060042 Bucharest, Romania

**Keywords:** polyurea, polyurethane, chain extenders, polymeric films, thermo-mechanical properties

## Abstract

The present work describes the synthesis of new versatile polyurea (PU) and polyurethane (PUR) matrices, including different chain extenders, which facilitate the design of distinct, tunable properties, and high-performance derivatives. These polymers can be used for various defense and security applications, such as coatings for ballistic protection, CBRN protection, binders for energetic formulations, etc. Combining aliphatic and aromatic molecules in PU or PUR structures enables the synthesis of polymers with improved and controllable thermo-mechanical properties. Thus, for polyurea synthesis, we utilized two types of polymeric aliphatic diamines and three types of aromatic chain extenders (1,1’-biphenyl-4,4’-diamine, benzene-1,2-diamine, and 1,2-diphenylhydrazine). An analogous method was used to synthesize polyurethane films by employing one polymeric aliphatic polyol and three types of aromatic chain extenders (benzene-1,3-diol, benzene-1,4-diol, and benzene-1,2,3-triol). Subsequently, various analytic techniques (Fourier transform infrared spectroscopy–attenuated total reflectance (FTIR-ATR), single cantilever dynamic mechanical analysis (DMA), thermogravimetric analysis (TGA), frequency-dependent shear modulus survey, tensile tests, water contact angle measurements, and scanning electron microscopy (SEM) with energy-dispersive X-ray analysis (EDX)) have been utilized to characterize the synthesized materials and to evaluate the influence of each chain extender on their final properties.

## 1. Introduction:

High-performance polymeric materials are the most viable alternative for designing “smart” defense technologies. However, developing new materials that assure the performance and safety requirements of defense and security products remains a significant challenge today.

Some of the most widely used elastomers are PU, PUR, and emerging PU–PUR materials [1,2]. PU and PUR are mainly used in defense and security applications as coatings for ballistic protection [3,4], protective garments for CBRN protection [5], or binders for energetic formulations [6]. Other uses for PU or PUR include adhesives, foams, construction materials, coatings, textiles, medical equipment, automobiles, and electronics [1,2,7,8]. Thus, due to their versatility, outstanding mechanical and chemical properties, and adaptability to specific designs, PU- and PUR-based materials have received much interest from related industries [7].

PU systems are formed by a step-growth polymerization reaction between polyisocyanates and amine-terminated compounds [9]. PUs, in general, are more resistant to high pH; they have superior thermal properties and higher melting points than other polymers [10]. PU derivates are widely used in various applications due to their remarkable properties, including high flexibility, high elasticity, impact resistance, light weight, heat resistance, low flammability, and good stability and durability [8,9,10]. PURs are formed by the step-growth polymerization reaction of polyisocyanates and compounds with hydroxyl functionalities, resulting in urethane linkages [11,12]. PURs possess numerous advantages, including high abrasion resistance, high load-bearing capacity (compared with other rubber materials), good damping properties, etc. [13].

The versatility of the formulations, the numerous possibilities to combine (macro)diols/(macro)diamines with (poly)isocyanates, and chain extenders contributes to the broad applicability of PU and PUR coatings. However, the appropriate choice of constituents and ratios between the reactants is crucial to the control of the macromolecular architecture and the desired properties for an application. PUs are elastomeric polymers with excellent performance due to their unique dual microphase structure (nanosegregated hard domains connected via flexible chains) and physical crosslinks [3,14]. PUR structures also comprise soft segments (SS) and hard segments (HS) [15]. Thus, the properties of PU and PUR systems can be tailored by adjusting the SS/HS ratio. In addition, chain extenders [16,17] may enable specific SS/HS patterns.

Chain extenders, curing agents, or crosslinkers are aromatic or aliphatic low-molecular-weight molecules (amine- or hydroxyl-terminated, with a functionality ≥ 2) that react with polyisocyanates to increase the volume of the segments with lower mobility (HS) to improve the final properties of the PU and PUR products [18]. In PU and PUR materials, chain extenders typically align themselves with the rigid and largely immobile hard segments inside the polymeric matrix [19]. In addition, higher functionalities lead to crosslinking between the tangled soft segments (SS) and the hard segments (HS) inside the PU and PUR matrices. Intermolecular bonds, established via hydrogen or van der Waals interactions, represent another essential aspect for PU and PUR properties. Thus, the interactions between the soft and hard segments in PU and PUR generate the desired characteristics, including elasticity, tensile strength, tear resistance, and elongation [20].

PU-based materials described in the literature are mainly obtained from commercial formulations: prepolymeric diamine components (oligomeric polyether diamines—e.g., Jeffamine^®^ [14,21,22] and Versalink^®^ [23,24]) and prepolymeric isocyanate components (aromatic isocyanates based on polymeric methylene diphenyl diisocyanate—e.g., Desmodur^®^ [25], Rubinate^®^ [26], or isophorone diisocyanate [27,28]). Commercial bicomponent prepolymeric formulations (macrodiol and polyisocyanate) are reported for PU-based materials (e.g., Vulkollan^®^ [20,29] and Multrathan^®^ [20]). Some studies also recommend a wide selection of chain extenders to improve the performances of PU matrices: primary amine chain extenders (e.g., isophorone diamine, ETHACURE^®^ 100) or secondary amine chain extenders (UNILINK^®^, CLEARLINK^®^, and JEFFLINK^®^) [30,31,32,33]. PUR chain extenders are usually diols (e.g., 1,4-butanediol and 1,3-butanediol) [20,34], but diamines are also reported as chain extenders for PUR matrices, leading to PU–PUR hybrids [18,35].

The main applications of PU and PUR are coatings (PU or PUR), sealants (PU or PUR), adhesives (PUR), elastomers (PU), and foams (PUR) [36,37].

As a part of modern and future marketing strategies, there is an increasing demand for multipurpose textiles [38]. A suitable coating can significantly increase the benefits of high-quality fabrics. Recent studies have been focused on the development of polymeric-based functional textiles. An important target has been the development of an optimal solution, because a coating layer covering textile substrates can affect the transfer of moisture, breathability, and the comfort characteristics [39]. Manjeet Jassal et al. demonstrated that the breathability of the PUR-coated cotton samples was directly influenced by the hydrophilic and hydrophobic components ratio [40]. Some publications have predicted special applications for PU or PUR textile coatings, and few are mentioned further. For example, Anwar Jahid et al. reported different types of shape-memory polyurethane coatings for cotton fabrics with remarkable water resistance and permeability properties for sportswear [41,42]. David De Smet et al. reported several types of polyurethane formulations as a waterproof coating for polyester textiles [36,43]. N. Raman Dhineshbabu et al. developed PUR coatings containing iron titanate nanoparticles for UV shielding [39]. Maryam Sharzaehee described oligomers with urea linkages deposited on jute fabric as a flame-retardant and antibacterial agent [44].

Numerous high-performance PU and PUR coatings have already been the subject of studies, but the area still requires significant attention. For example, Stephane Giraud et al. reported intumescent flame-retardant polyurea coating for textiles [45]. Some recent achievements, relevant to the defense industry, are cited further. Jihyun Choi et al. developed a polyurethane-based protective cloth against chemical warfare agents (CWAs) [46]. Razvan Petre et al. reported polyurea-MWCNT nanocomposite coatings deposited on textile gloves which offered over 24 h of protection, for a contamination density of 50 g/m^2^, against persistent CWAs (mustard gas, soman, and Vx) [5]. In addition to CBRN protection applications, ballistic protection is another field in which PU–PUR materials are valuable. PU coating could significantly increase the resistance of conventional ceramic/metal armor to create a new armor system without adding extra weight [47]. In one of our previous studies [48], we demonstrated that PU–PUR-coated aluminum plates maintained their integrity at impact with a projectile. Many other studies emphasize the advantages of high-performance PU–PUR materials for ballistic protection applications, and a few examples are provided below. Jamil et al. [49] demonstrated that increasing the thickness of the PUR core in the sandwich panels increased the blast resistance of the structure. Yongqing Li et al. [50] described the influence of the spraying strategy on the dynamic response of polyurea-coated metal plates to localized air blast loading.

Unfortunately, only a small number of studies are available in the literature related to the chemistry of the chain extenders employed in PU and PUR systems. Manorama Tripathi et al. [51] studied the effect of the length of three aliphatic chain extenders (ethylene diamine, Jeffamine^®^ D-230, and Jeffamine^®^ D-400) on the properties of the resulting PU, in comparison with two aromatic chain extenders (3,5-diethyltoluene-2,4-diamine and 3,5-diethyltoluene-2,6-diamine). However, recent research has revealed that aromatic chain extenders are significantly more reactive than their aliphatic equivalents, resulting in noticeably shorter gel times [3,52].

Capitalizing on the versatility of PU and PUR and their compatibility with various aromatic chain extenders, this study aimed to develop PU and PUR coatings, with adjustable compositions, that may provide the appropriate features for specific defense industry products. Thus, different formulations of PU and PUR were synthesized to investigate the effect of the chain extenders on the properties of the films. The structure–property relationship of PU and PUR can be adjusted to obtain specific macromolecular architectures by introducing low-molecular-weight molecules in the design of PU and PUR. To the best of our knowledge, this is the first study that investigates the potential effect of distinct aromatic chain extenders on the final characteristics of PU and PUR coatings, which might prove suitable for defense applications. To obtain high-performance PU and PUR films, the current research describes the synthesis and characterization of novel formulations based on three types of aromatic chain extenders for each polymer class considered: polyurea and polyurethane.

## 2. Materials and Methods

### 2.1. Materials

*Chain extenders*: 1,1′-biphenyl-4,4′-diamine (BZ, benzidine), benzene-1,2-diamine (o-PhDA, o-phenylenediamine), 1,2-diphenylhydrazine (di-PhHA, N,N′-Diphenylhydrazine), benzene-1,3-diol (RZ, Resorcinol), benzene-1,4-diol (HQ, hydroquinone), and pyrogallol (Py, 1,2,3-trihydroxybenzene) from Sigma Aldrich, St. Louis, MO, USA were used as received; *prepolymers:* poly (propylene glycol) bis(2-aminopropyl ether) (PPG2000, Mn ≈ 2000 Da, Sigma Aldrich, St. Louis, MO, USA), Poly (propylene glycol) bis(2-aminopropyl ether) (PPG4000, Mn ≈ 4000 Da, Sigma Aldrich, St. Louis, MO, USA), diphenylmethane-4,4′-diisocyanate (MDI, technical product Desmodur^®^ 44V20L, -NCO content 31.5%, Covestro, Leverkusen, Germany), and acetone (Ac., Sigma Aldrich St. Louis, MO, USA) were used as received. Setathane^®^ D1160 (SET, technical product, slightly branched castor oil-based polyol, hydroxyl content 5.4%, Allnex, Brussels, Belgium) was vacuum dried for 24 h at 50 °C before being employed for polyurethane films synthesis.

### 2.2. Methods

#### 2.2.1. Synthesis

Polyurea synthesis (PU)

Two solutions (A and B, Table 1 and Figure S1—simplified scheme) were employed to synthesize each type of polyurea film, utilizing the casting method described below. The first step involved the dissolution of PPG 2000 or 4000 in acetone, followed by the dissolution of BZ, o-PhDA, or di-PhHA in the same solution (A). The second solution (B) was obtained by dissolving MDI in the same amount of acetone (15 mL). The molar ratio between amino groups and isocyanate groups was constantly 1:1. After vigorously mixing solutions A and B for approximately 15 s, the mixture was transferred to a Petri dish on a flat surface and kept at 25 °C and 50–55% relative humidity. Films with a thickness of about 0.5 mm resulted after complete curing.

Polyurethane synthesis (PUR)

An analogous synthesis path was used to obtain polyurethane films, utilizing the casting method described below. Thus, two solutions (A illustrated in Table 2 and Figure S2—simplified scheme and B (containing MDI)) were vigorously stirred for approximately 15 s. The molar ratio between hydroxyl groups and isocyanate groups was 1:1. Each mixture was subsequently transferred to a Petri dish, placed on a flat surface, and kept at 25 °C (50–55% relative humidity). Films with a thickness of approximately 0.7 mm resulted after complete curing.

#### 2.2.2. Textile Coatings with PU and PUR

Textile specimens (2 cm × 5 cm) from the army combat uniform were immersed in two of the Petri dishes containing the reaction mixtures PU-2.0-Bk and PUR-S0-Bk to investigate the possibility of employing herein-reported PU and PUR coatings for CBRN protection garments. These coatings (and the corresponding neat films) were further analyzed via SEM-EDX. These trials serve as preliminary investigations for developing PU and PUR-coated textiles designed for CBRN protection purposes.

#### 2.2.3. Characterization

Perkin Elmer Spectrum Two (Perkin Elmer, Waltham, MA, USA) with a Pike MiracleTM ATR modulus was used to obtain FTIR spectra of PU and PUR films (wavenumber range: 550–4000 cm^−1^, resolution: 1 cm^−1^, data averaging: 32 scans). Single cantilever mode was utilized for the dynamic mechanical analysis (DMA) of PU and PUR films. These tests were performed in similar conditions for PU and PUR films (samples dimensions: 36 mm × 10 mm × 0.5–0.7 mm, frequency: 1 Hz, temperature ramp: −90 °C to +50 °C, heating rate: 5 °C/min, cooling: liquid nitrogen) on a Discovery DMA 850 apparatus from TA Instruments (TA Instruments, New Castle, DE, USA). On the same Discovery DMA 850 instrument, the shear-sandwich clamp was mounted to evaluate the frequency-dependent shear modulus of PU and PUR films (frequency range: 10 Hz–100 Hz, oscillation amplitude: 20 µm, 25 °C); method adapted from ASTM D 945-92 Standard Test Methods for Rubber Properties in Compression or Shear. Afterward, a tensile test clamp was installed and calibrated for uniaxial deformation investigations on the same Discovery 850 DMA apparatus. Tensile tests were conducted on rectangular specimens of PU and PUR (measuring 50 mm × 8 mm × approximately 0.5–0.7 mm) at 5 mm/min at 25 °C. Five specimens from each material were subjected to tensile tests, and the mean values were reported. Tensile tests were adapted following ASTM D-638-Standard Test Method for Tensile Properties of Plastics for 25 mm gauge length. The thermogravimetric analyses (TGA) of the catalysts were performed using a Netzsch TG 209 F3 Tarsus equipment considering the following parameters: nitrogen atmosphere flow rate 20 mL min^−1^; samples mass ~4 mg; temperature range: room temperature −900 °C; and heating rate: 10 °C min^−1^ in an alumina (Al_2_O_3_) crucible. For static contact angle measurements on dried films, a KSV CAM 200 apparatus was used. Distilled water drops (20 µL) were placed on the samples and analyzed. Each contact angle was measured within 10 s to ensure that the droplet did not soak into the film. The contact angles reported were the average of five measurements on different parts of each film. The morphology of the synthesized materials and the distribution of their main elements were investigated through SEM-EDX using a Tescan Vega II LMU SEM instrument (TESCAN, Brno, Czechia) at 10 keV acceleration voltage, under a high vacuum. The samples were gold-sputter-coated to ensure proper data acquisition.

## 3. Results

Polyurea is a versatile polymer, commonly possessing a phase-segregated microstructure [53]. It has been described in the literature that, as the curing temperature increases, the degree of microphase separation increases, and the compatibility of the soft and hard segments decreases [54].

As illustrated in Figure S1, the long-chain polyethers prepolymer represent the soft part of the polyurea network and provide its extensibility. At the same time, the hard domains established by MDI (F ≥ 2) serve as chemical crosslinks. In addition, the H-bonded urea linkages also contribute to matrix reinforcement and physical crosslinks [51]. Likewise, numerous studies on polyurethane microphase separation have recently been published [15]. As might be expected, this microphase separation significantly influences the performances of PU and PUR.

Thus, in addition to the classical prepolymers (PPG and MDI), we introduced three types of chain extenders to synthesize polyurea films (Figure S1). The first molecule employed as a chain extender was 1,1′-biphenyl-4,4′-diamine (benzidine), a biphenyl amine consisting of two covalently bonded benzene rings, laterally substituted by two amino groups (at 4 and 4′, respectively). This molecule was chosen to increase the amount of HS and to ascertain how it might impact the final properties of PU.

The second chain extender was a smaller molecule, o-phenylenediamine, which was employed to determine the influence of the vicinal amino groups, present in phenylenediamine, on the behavior of the hard domains of PU. Since it is well known that secondary amines slow down the curing speed of polyurea elastomeric coatings, the third chain extender chosen for the PU synthesis was hydrazobenzene. This chain extender is a diphenyl derivative of hydrazine, thus a molecule comprising two secondary amines substituted with aromatic moieties. It was employed to synthesize the third type of PU coating to ascertain its influence on the characteristics of the polymeric matrix.

In the same way, for PUR synthesis, three distinctly substituted aromatic rings were utilized as chain extenders (Figure S2—resorcinol, hydroquinone, and pyrogallol) to establish the modification of the performance characteristics of the PUR networks.

The initial stage of this research involved the synthesis of PU and PUR films, respectively. To efficiently dissolve the chosen chain extenders and to obtain homogeneous PU and PUR films, we used acetone as the solvent. Subsequently, thin films were produced by the casting method. In PU synthesis, the prepolymers employed exhibited a high reactivity; thus, compared with PUR synthesis, higher amounts of acetone were necessary to allow good mixing of the components before transferring the reaction mixtures on the Petri plates.

Thus, twelve distinct types of PU (eight formulations) and PUR (four formulations) films were obtained and subjected to specific analytical investigations to evaluate the physicochemical properties of the materials.

**Figure 1:**
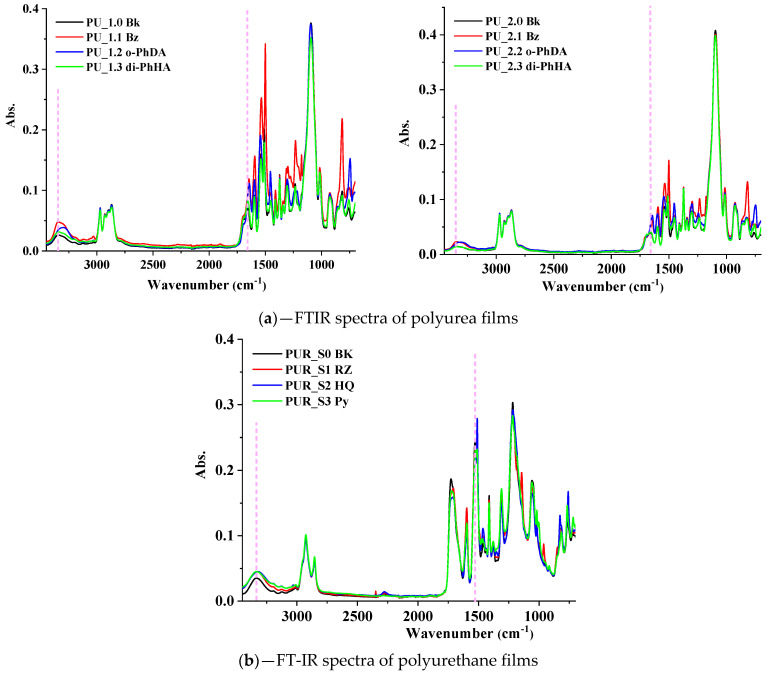
FTIR spectra of PU and PUR films.

The FTIR analysis was carried out to ascertain the formation of the urea/urethane groups. Figure 1 illustrates the FTIR spectra of polyurea (Figure 1a) and polyurethane (Figure 1b) films obtained. FT-IR analysis revealed the distinctive spectral characteristics of the synthesized materials. Thus, PU matrices (Figure 1a), carbonyl (1657 cm^−1^), and N-H (3343 cm^−1^) stretching bands seemed to slightly shift to lower wavenumber values due to the newly established intermolecular or intramolecular hydrogen bonding, demonstrating that aromatic extended regions of PU and PUR exhibited stronger H-bonding compared with highly aliphatic structures [51]. PU samples containing o-PhDA showed a larger “red-shift” [51,55], indicating stronger H-bonding. In PUR spectra (Figure 1b), the urethane N-H stretching band, visible around 3335 cm^−1^, is slightly shifted to 3333–3320 cm^−1^ for the PUR matrices containing the aromatic chain extenders, suggesting the formation of supplementary hydrogen bonds. The presence of 1727 cm^−1^ (C=O stretch of urethane) and 1059 cm^−1^ (C-O-C stretch) peaks confirmed the formation of the urethane linkage. The intensity and the position of the peak that can be assigned to νC=C from the aromatic rings (~1539 cm^−1^ in PU or 1527 cm^−1^ in PUR) shifted depending on the aromatic chain extenders introduced in PU and PUR matrices, suggesting the distinct types of interactions established between their components. In addition to the HS/SS ratios, hydrogen bonding also plays an essential role in determining the final properties of PU–PUR matrices [55]; thus, these strong H-bonds, confirmed via FT-IR, may promote obtaining the optimal properties for PU–PUR coatings designed for the defense industry applications.

**Figure 2:**
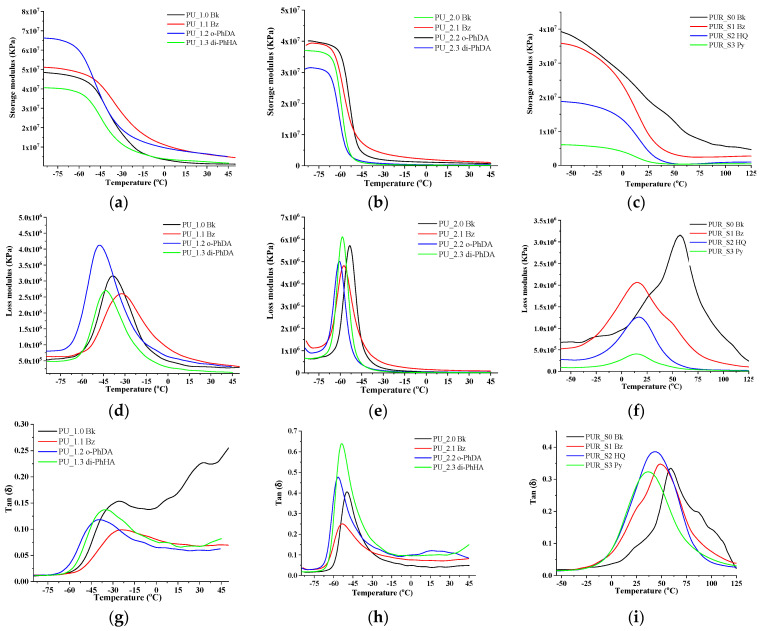
Storage modulus (**a**–**c**), loss modulus (**d**–**f**), and tan delta (**g**–**i**) of PU and PUR films.

Dynamic mechanical analysis was performed for PU and PUR films to examine the changes in their mechanical properties under periodic stress as temperature increased. Thus, experimental investigations of PU and PUR were carried out in single cantilever mode temperature scan. The variation of storage, loss modulus, and tan delta for PU and PUR formulations, as a function of temperature, are presented in Figure 2. As can be observed, the motions associated with the SS of the polymeric matrices generated one relaxation peak, visible in all of the formulations. In addition, the storage modulus (E’) increased for the PU_1 series of samples containing BZ and o-PhDA, indicating an increase in the stiffness of these materials, probably due to the restricted mobility of *sp^2^*-hybridized carbon atoms from the HS region. In contrast, for the PU samples containing di-PhHA, E’ decreased, since *sp^3^* hybridization of nitrogen atoms from di-PhHA ensures an internal free rotation around their N-N bond [56], thus leading to higher mobility of the polymeric chains connected to this moiety. Therefore, the increase in the amount of relatively stiffer *sp^2^*-hybridized aromatic groups in the PU matrix was reflected through higher storage modulus values. This was no longer the case for the PU_2 or PUR samples, whose longer aliphatic chains caused a decrease in E’ values. Compared with its storage capacity, a higher tan delta (damping loss factor) indicates that a material has a relatively higher potential for energy dissipation. This information is valuable for PU–PUR matrices designed for defense products. As can be observed from Figure 2g,h, samples containing di-PhHA, especially the ones with longer aliphatic chains, displayed remarkable energy dissipation abilities. Accordingly, they led to a higher tan delta than the other chain extenders employed. In fact, excepting the samples with BZ, the other PU specimens displayed higher energy dissipation efficiencies. Tan delta values for the PUR series shifted towards lower temperatures (Figure 2i) after the introduction of chain extenders.

DMA analysis also allowed the identification of the glass transition temperatures of the synthesized materials. Table 3 summarizes the glass transition temperatures obtained for PU and PUR coatings based on the temperature of the tan delta maxima [57]. It can be observed that the PU samples containing o-PhDA and di-PhHA possessed the lowest T_g_ values. The PU_2 sample series displayed lower Tg values, since it contains longer aliphatic chains than the PU_1 sample series. The position of the amine functionality or the presence of pending phenyl groups (in the case of di-PhHA) leads to a modification of the intermolecular chain interaction and its packing (Scheme S1). A lower molecular chain packing leads to a lower T_g_ value. The addition of the aromatic chain extenders in the case of PUR leads to a decrease in the Tg. The lowest T_g_ was obtained for PUR samples when employing Py as a chain extender, which signifies the highest chain mobility, which could be attributed to the steric hindrance between the OH groups. Therefore, not all OH groups are reacted, leading to an unexpected behavior of the PU containing this chain extender.

Given that superior thermal resistance is necessary for defense applications, the thermogravimetric analysis of PU and PUR films aimed to discover their decomposition pattern. The results obtained from TGA analysis are illustrated in Figure S3 and summarized in Table 4. As can be observed, all the PU and PUR coatings possess remarkable thermal stability, up to 257 °C. Compared with the other samples from the same PU series, PU samples containing o-PhDA displayed a two-stage degradation profile (Figure S3) and noticeably lower thermal stability. All the other PU samples exhibited similar thermal behavior, consisting of a singular degradation peak with the maximum situated between 312–328 °C. In contrast, the PUR series displayed four peaks on DTG plots but, at the same time, similar thermal behaviors as the PUR samples (decomposition peak maximum between 323–333 °C). Compared with blank samples, the decomposition process began slightly earlier for the samples containing the aromatic chain extenders, because it is well known that the aromatic moieties tend to degrade earlier than the aliphatic polyether chains [55]. Nonetheless, the decomposition peak maximum was relatively similar for analogous samples.

**Figure 3:**
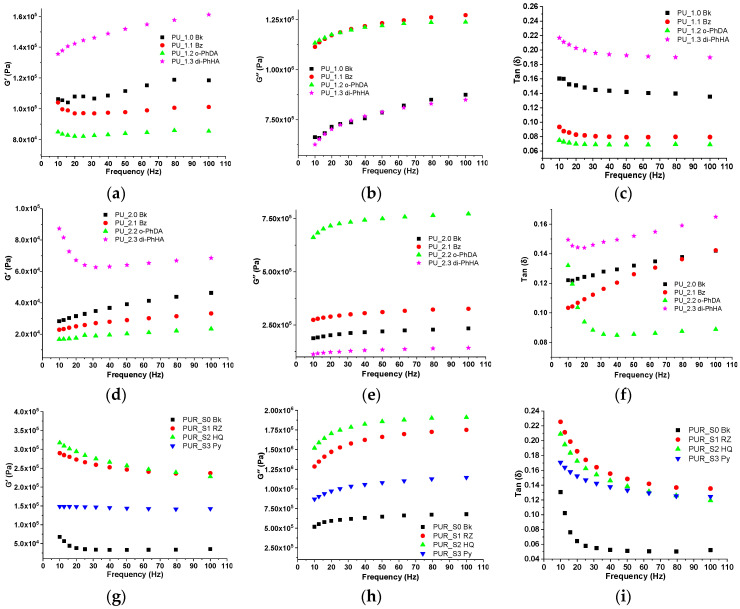
Shear storage modulus (**a**,**d**,**g**), shear loss modulus (**b**,**e**,**h**), and tan delta (**c**,**f**,**i**) vs. frequency for PU and PUR films.

Figure 3 shows the results obtained in the “shear-sandwich” DMA set-up. Shear storage modulus, shear loss modulus, and tan delta are herein emphasized. For determining the viscoelastic properties of polymeric materials, oscillatory shear is recurrently used. Two identical PU_1, PU_2, or PUR specimens of the same shape and size (11 mm × 11 mm) were “sandwiched” between two fixed metallic plates and a central oscillating plate in “shear-sandwich geometry”. A viscoelastic material responds to an external stress with an elastic deformation (storing energy) and a viscous flow (dissipating energy) [58]. All oscillatory shear experiments were conducted at small stress amplitudes to remain within the linear viscoelastic region. As can be observed, the viscoelasticity of PU and PUR samples varies with frequency. In the PU_1 series, the shear storage modulus was higher for the sample containing di-PhHA. Because shear occurs at relatively low frequencies, low-frequency media have a high capacity to maintain their initial strength [59].

The shear rate increases with higher frequency, enhancing input on polymer chains. From the PU_1 series, samples containing o-PhDA or BZ, the storage shear modulus appeared less dependent on frequency, probably due to the restricted mobility of their components. In contrast, the PU_2 sample series, being more elastic, displayed a much more frequency-dependent behavior. Except for PU_2—diPhHA, higher slopes for the G′ plots of the other specimens from the PU_2 series are explained by the higher relaxation times required by these polymeric networks. In the case of the PUR series, the coatings containing the aromatic chain extenders exhibited a stiffening effect visible through the higher values of the storage shear modulus obtained for these specimens. The storage modulus of PUR_Py seemed to be almost frequency-independent. Tan delta, representing the ratio of the moduli (G″/G′), indicates the relative degree of damping of the material.

When a material is dynamically loaded, its inherent damping is characterized by the loss factor. In defense applications, damping is essential in preventing early structural damage brought by external stimuli. Measuring the linear viscoelastic properties helps understand the relationship between molecular structure and product performance. The damping behavior of PU and PUR specimens was frequency-dependent, as shown in Figure 3c,f,i. The values obtained for tan delta were in accordance with their composition.

**Figure 4:**
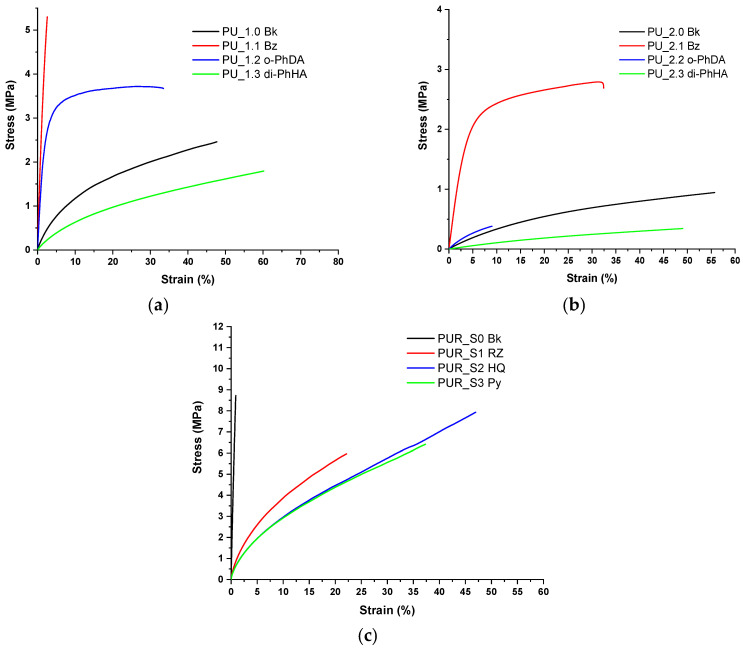
Comparative results for tensile test: (**a**) PU_1 series, (**b**) PU_2 series, and (**c**) PUR series.

Tensile testing is a fundamental technique for assessing the mechanical properties of polymers and an indispensable tool for materials science. The PU–PUR films were subjected to tensile testing. The results are summarized in Figure 4 and detailed in Figure S4. Among the chain extenders selected for PU coatings, BZ registered the highest tensile stress values but also low strain values. The PU samples containing o-PhDA withstood higher stress than the analogous blank samples, but they lacked elasticity. PU samples containing di-PhHA exhibited the highest elasticity, ensured by the mobility provided by this chain extender to these polymeric systems. PU_1.3 di-PhHA registered higher strain values than the analogous blank PU sample (PU_1 Bk). For the PUR sample series, the introduction of the chain extenders ensured the improvement of the strain values. Their influence on stress–strain plots is in accordance with their chemical structure and the position of the hydroxyl substituents on each aromatic ring introduced. To summarize the results obtained from tensile tests, we can affirm that the aromatic chain-extended PU and PUR materials possess a more rigid polymeric matrix due to aromatic rings introduced in their crosslinked structure. Higher tensile strength resulted from this behavior, except for the samples containing di-PhHA, which ensured greater mobility.

Water contact angles measured for the PU and PUR films, detailed in Table S1, indicate they possess moderate hydrophilic surfaces (contact angles between 41 and 70 degrees for PU and contact angles between 36 and 73 degrees for PUR). The hydrophilicity for PU series increases with the increase of PPG-derivate molecular weight (lower contact angles for PU2 series), while for the PUR samples, the increase in the hydroxyl groups leads to a higher hydrophilicity (lower contact angle). PU and PUR designed for CBRN protection coatings may benefit from examining this parameter, even though the water contact angle is insufficient to fully characterize the adhesion of potential contaminants. Additional research regarding the permeation of the toxic agents through the polymeric film is recommended for future dedicated studies.

**Figure 5:**
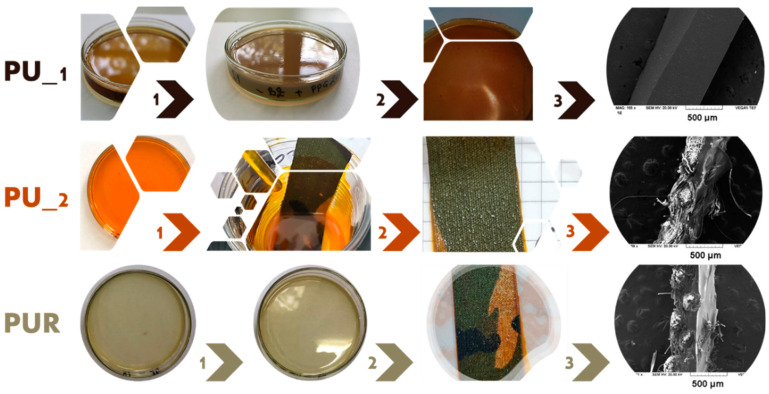
PU and PUR coatings obtained through the casting method: step 1—liquid reactive formulations (solvent–acetone); step 2—textile impregnation/sol–gel transition and curing process; and step 3—cured film/coating.

**Figure 6:**
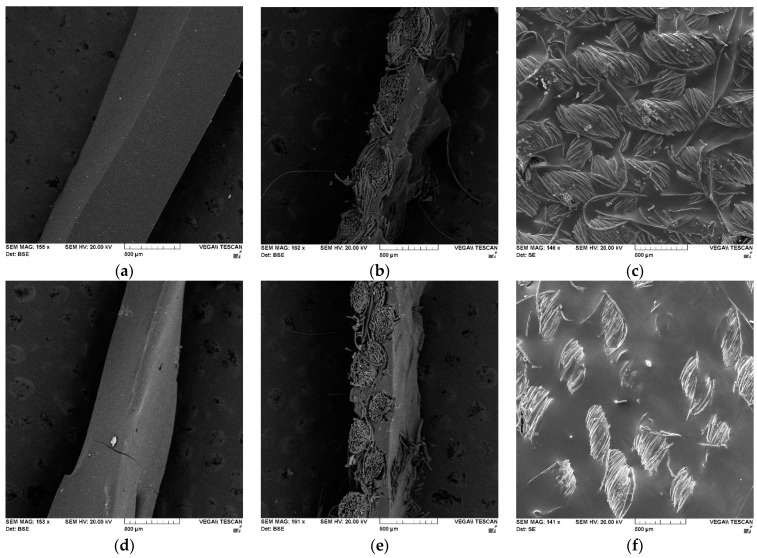
SEM images of PU and PUR films and PU and PUR textile coatings: (**a**)—neat PU; (**b**)—PU-coated textile, side view; (**c**)—PU-coated textile, front view; (**d**)—neat PUR; (**e**)—PUR-coated textile, side view; and (**f**)—PUR-coated textile, front view.

The last part of the experimental study consisted of a preliminary evaluation of the possibility of coating combat uniform textile specimens with PU and PUR films for upcoming auto-decontamination applications designed for the defense industry. It is essential to mention that these preliminary studies are continued presently by introducing some active ingredients (against chemical/biological agents) inside the polymeric matrix. Still, the first step for this type of application is to investigate the compatibility between the textile substrate and PU and PUR coatings applied. Thus, two of the PU and PUR formulations were utilized for textile coatings to examine the possibility of employing these polymeric films for future CBRN protection applications (PU_2.3 di-PhHA and PUR-S3-Py-Figure 5). For ease of application of the polymer coating on the textile specimen, PU 2.3 di-PhHA was chosen due to its high flexibility and longer curing times. In addition to this, PUR-S3-Py was selected for textile coating because it displayed the highest flexibility among PUR series specimens. The spray coating method (Figure S7) offers several industrial advantages compared to casting method, such as the facile coverage of larger areas. However, the casting method is still the most feasible for laboratory-scale experiments. Figure 5 explains the main steps of obtaining PU and PUR films or textile coatings, respectively, through the casting method. Prior to deciding which specimens should be used for coating textiles, neat films from each type of sample were obtained using the casting method. PU_1.1-BZ film, illustrated in the first row of Figure 5, serves as an example. However, because it was a rigid film, it was not chosen for the survey on textile. Contrarily, PU 2.3 di-PhHA appeared to be a proper option for this type of application, because it was the most flexible of all PU series specimens. For this reason, PU_2.3 di-PhHA was chosen to be deposited on textile specimens from combat uniforms (see Figure S5 for uncoated specimens), as can be observed in the middle row of Figure 5. Additionally, the most flexible sample from PUR series, PUR-S3-Py (bottom-row, Figure 5), was also deposited on the same type of textile specimens. Finally, the SEM-EDX survey was used to examine the morphology of the neat polymeric films and the aspect of the textile specimens coated with PU or PUR. The most relevant results are illustrated in Figure 6, and additional images are presented in Figure S6.

As can be observed from Figure 6 and Figure S6, the neat PU and PUR films obtained via the casting method display a homogenous morphology, possessing a smooth surface, without visible pores. Figure 6b,e represents a side view of the PU- and PUR-coated textile specimens. Because the PU formulation had a lower viscosity than PUR (when the fabric was immersed in the reaction mixture), the PU produced a thinner textile coating than PUR.

## 4. Conclusions

Polyurea and polyurethane (PU and PUR) are versatile polymers with a high potential for applications in the defense industry. In this study, various PU- and PUR-based formulations containing a set of aromatic chain extenders were obtained and characterized to assess their key characteristics and identify the potential applications they might be suitable for based on their specific features. In addition, the adjustable compositions could provide the required functionality for specific defense industry products. Specialized analytic tools were employed to characterize the synthesized materials and to highlight their performances. FTIR analysis confirmed the formation of the characteristic urea and urethane bonds, respectively. The length of the aliphatic chains and the characteristics of the aromatic chain extenders both significantly impacted the mechanical and thermal properties of the PU and PUR coatings. For the PU coatings, it was observed that o-PhDA had the most significant negative impact on their thermal and mechanical properties, while di-PhHA induced remarkable thermal resistance and higher elasticity. BZ had a stiffening effect on the PU films. For PUR coatings, the three chain extenders rendered higher storage and shear loss modulus, thus better damping capacity.

The PU and PUR coatings should meet specific requirements, depending on the application for which they are intended, including high mechanical resistance, high thermal resistance, and high resistance to toxic vapors or droplets. The specimen we studied, PU 1.1-BZ, seems to be the most suitable for ballistic protection applications due to its demonstrated high mechanical and thermal resistance. As shown in our previous studies [33,48], materials with a high capacity for absorbing and dissipating energy can substantially improve the ability to mitigate the effect of shockwaves on the metallic structures on which PU is applied. In contrast, the high flexibility and the homogenous coatings containing di-PhHA (especially PU_2.3 di-PhHA) make them ideal for textile coatings.

Future research will examine the viability of using these polymeric films for CBRN protection applications and other functional composites for diverse applications.

Technology will soon be able to produce uniform nanoscale components and incorporate them into complex macromolecular structures, giving researchers new levels of control over the physical and chemical characteristics of the constituents of macroscopic materials.

## Figures and Tables

**Table 1:** Components for PU synthesis.

Solutions:	A					
Sample Code	PPG2000 [g]	PPG4000 [g]	BZ [g]	o-PhDA [g]	di-PhHA [g]	Ac. [mL]
PU-1.0-Bk	1.65	-	-	-	-	15
PU-1.1-BZ	1.65	-	0.6	-	-	15
PU-1.2-o-PhDA	1.65	-	-	0.35	-	15
PU-1.3-di-PhHA	1.65	-	-	-	0.6	15
PU-2.0-Bk	-	3.3	-	-	-	15
PU-2.1-BZ	-	3.3	0.6	-	-	15
PU-2.2-o-PhDA	-	3.3	-	0.35	-	15
PU-2.3-di-PhHA	-	3.3	-	-	0.6	15

**Table 2:** Components for PUR synthesis.

Solution:	A				
Sample Code	SET [g]	RZ [g]	HQ [g]	Py [g]	Ac. [mL]
PUR-S0-Bk	2	-	-	-	15
PUR-S1-RZ	2	0.2	-	-	15
PUR-S2-HQ	2	-	0.2	-	15
PUR-S3-Py	2	-	-	0.23	15

**Table 3:** Glass transition temperatures * of PU and PUR, obtained from DMA analysis.

Sample Code	T_g_ (°C)	Sample Code	T_g_ (°C)	Sample Code	T_g_ (°C)
PU-1.0-Bk	−25.6	PU-2.0-Bk	−49.6	PUR-S0-Bk	58.9
PU-1.1-BZ	−24.0	PU-2.1-BZ	−53.3	PUR-S1-RZ	47.9
PU-1.2-o-PhDA	−38.7	PU-2.2-o-PhDA	−56.9	PUR-S2-HQ	43.2
PU-1.3-di-PhHA	−34.8	PU-2.3-di-PhHA	−53.5	PUR-S3-Py	37.0

* T_g_ evaluation was based on the temperature of maximum tan delta (Figure 2g–i), according to ref. [57].

**Table 4:** Decomposition temperatures of PU and PUR, obtained from TGA analysis.

Sample Code	T_10%_ (°C)	T_d_ (°C)	Sample Code	T_10%_ (°C)	T_d_ (°C)	Sample Code	T_10%_ (°C)	T_d_ (°C)
PU-1.0-Bk	326.7	376.7	PU-2.0-Bk	328.4	378.5	PUR-S0-Bk	309.3	329.3
PU-1.1-BZ	312.8	367.8	PU-2.1-BZ	328.6	379.6	PUR-S1-RZ	297.9	332.9
PU-1.2-o-PhDA	257.6	375.1	PU-2.2-o-PhDA	277.3	379.8	PUR-S2-HQ	298.5	323.5
PU-1.3-di-PhHA	321.5	371.5	PU-2.3-di-PhHA	317.1	379.7	PUR-S3-Py	297.8	325.3

T_10%_ = decomposition onset temperature (measured at 10% weight loss); T_d_ = the maximum decomposition temperature related to the DTG peak (Figure S3), where the weight loss rate reached the maximum value.

## Data Availability

The data presented in this study are available on request from the corresponding author.

## Supplementary Materials

The following supporting information can be downloaded at: https://www.mdpi.com/article/10.3390/polym15030756/s1. The following Supplementary Materials, including Figures S1 and S2—The components of polyurea and polyurethane films; Scheme S1—Schematic representation of PU chains mobility—Tg variation explanation; Figure S3—TGA (a,c,e) and DTG (b,d,f) curves for PU and PUR films; Table S1—Water contact angle measurements for PU and PUR films; Figure S4—Tensile tests; Figure S5—SEM images of uncoated textile specimens; Figure S6—SEM—EDX mapping of the coated textile specimens; Figure S7—Spray forming advantages for ballistic protection applications.
