# Supplementary material for: Effect of Aromatic Chain Extenders on Polyurea and Polyurethane Coatings Designed for Defense Applications
Source: Polymers (Basel). 2023 Feb 2;15(3):756. doi: 10.3390/polym15030756 (PMC9920908; doi:10.3390/polym15030756)
Supplement: Supplementary file 1 [file polymers-15-00756-s001.zip › polymers-2150028-supplementary.pdf]

polymers
MDPI

Supplementary Material

Effect of Aromatic Chain Extenders on Polyurea and Polyurethane Coatings Designed for Defense Applications

Gabriela Toader [1,†], Andreea Elena Moldovan [1,†], Aurel Diacon [1,2,*], Florin Marian Dirloman [1,2,*], Edina Rusen [2], Alice Podaru [1,2], Traian Rotariu [1], Raluca Elena Ginghina [3] and Oana Elisabeta Hoza [2,4]

1 Military Technical Academy "Ferdinand I", 39–49 George Cosbuc Boulevard, 050141 Bucharest, Romania
2 Faculty of Chemical Engineering and Biotechnologies, University of Bucharest, 1–7 Gh. Polizu Street, 011061 Bucharest, Romania
3 Research and Innovation Center for CBRN Defense and Ecology, 225 Oltenitei Ave., 041327 Bucharest, Romania
4 Faculty of Material Science and Engineering, University of Bucharest, 313 Splaiul Independentei, District 6, 060042 Bucharest, Romania
* Correspondence: aurel_diacon@yahoo.com (A.D.); florin.dirloman@mta.ro (F.M.D.)
† These authors contributed equally to this work.

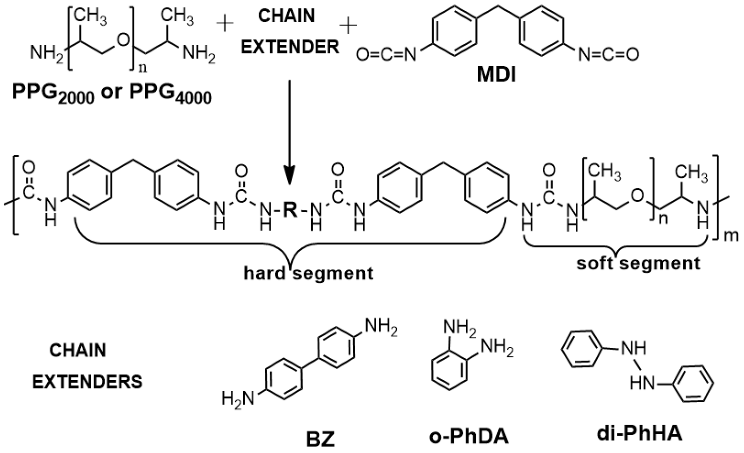

Figure S1. The components of polyurea films.

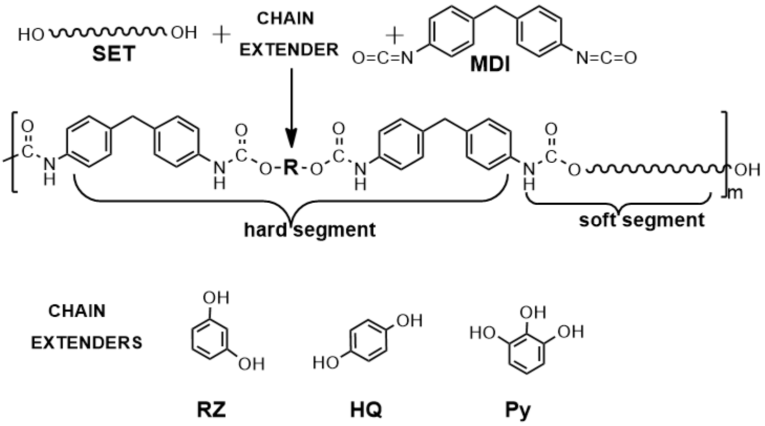

Figure S2. The components of polyurethane films.

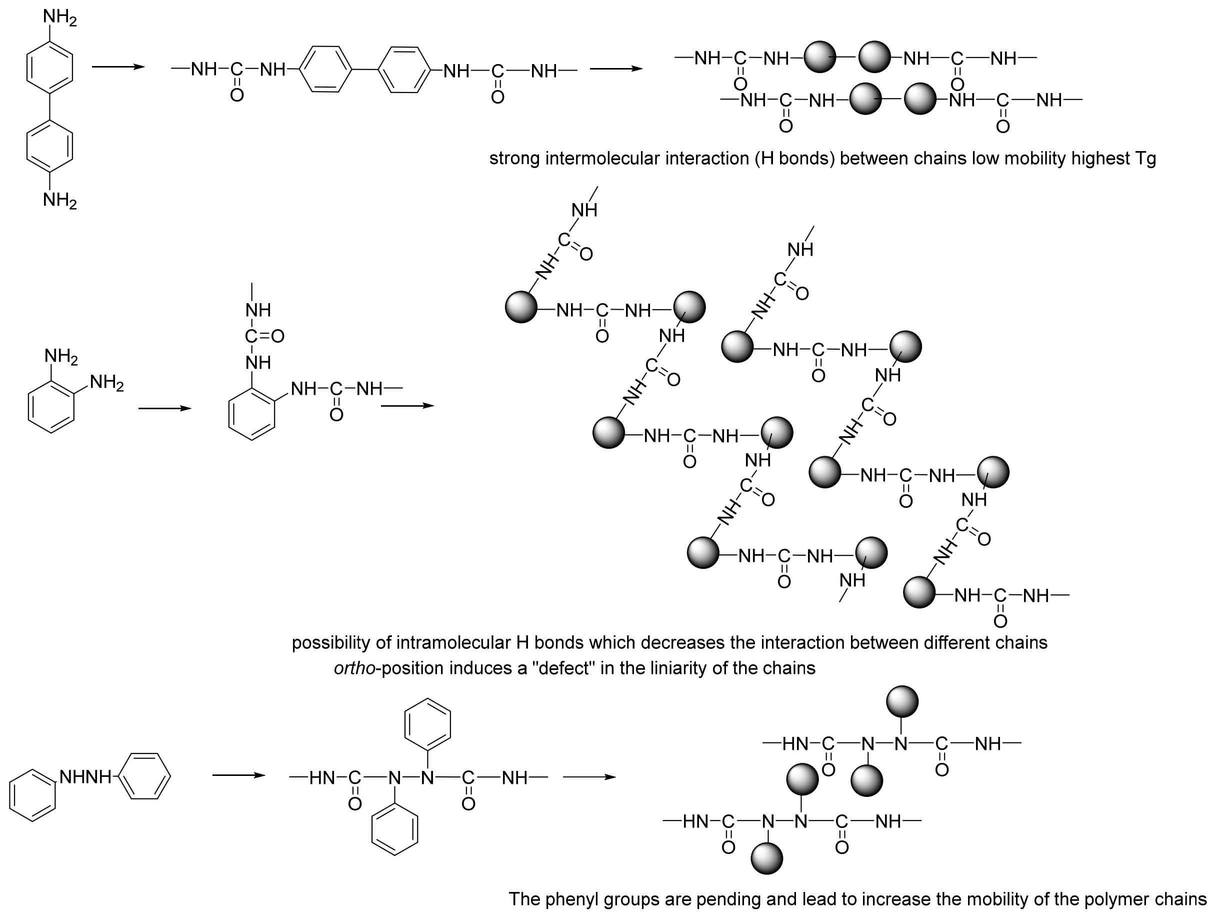

Scheme S1. Schematic representation of PU chains mobility – T_g variation explanation.

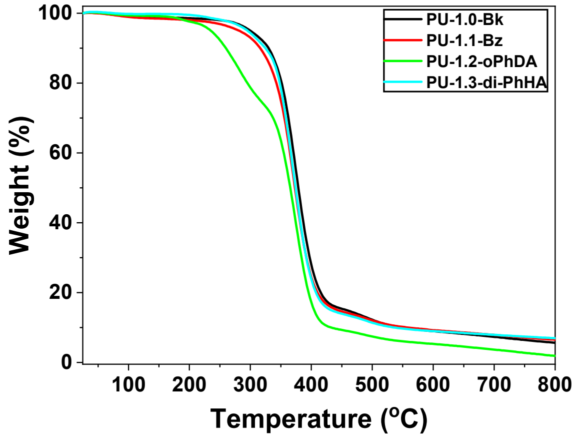

a

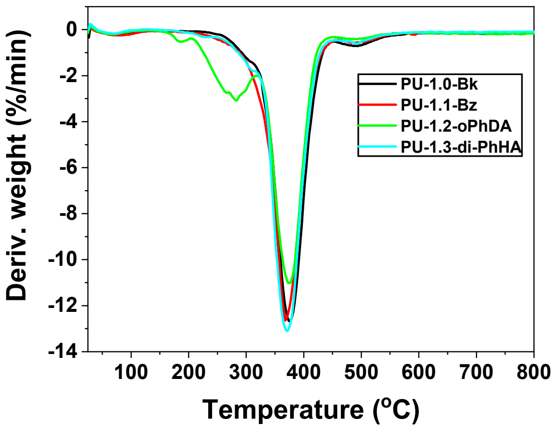

b

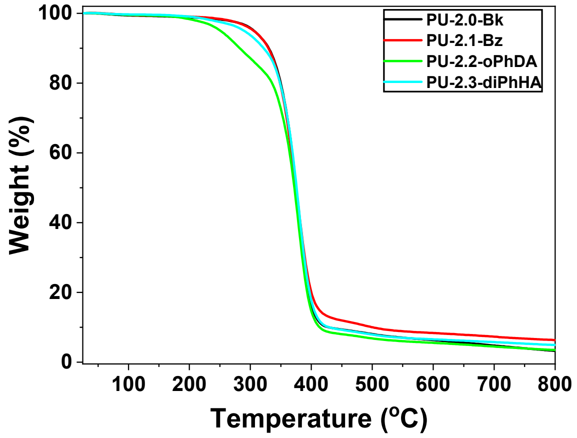

c

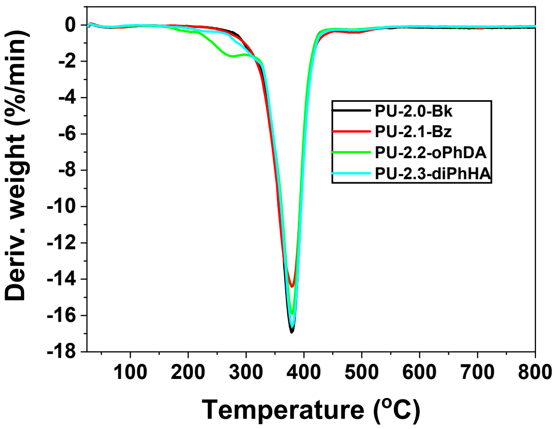

d

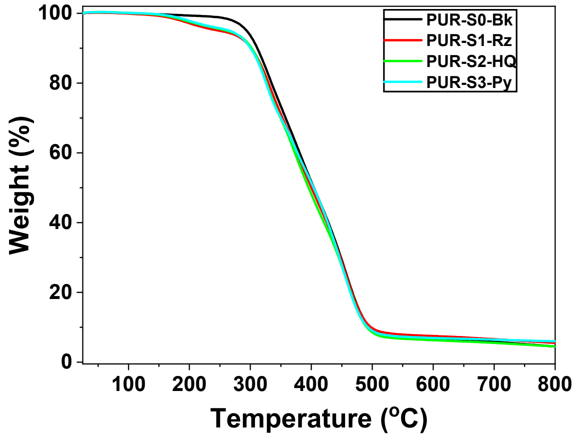

e

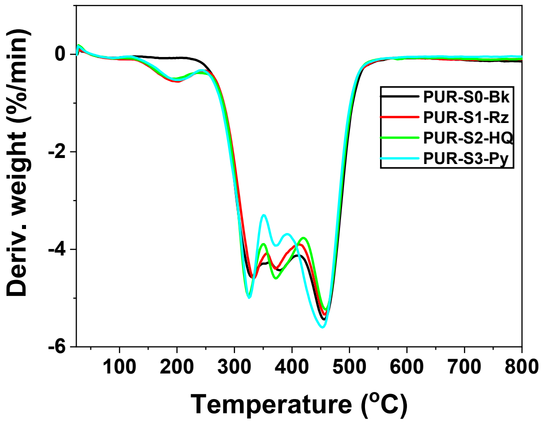

f

Figure S3. TGA (a, c, e) and DTG (b, d, f) curves for PU and PUR films.

Table S1. Water contact angle measurements for PU and PUR films.

Sample code	WA (°)	Sample code	WA (°)	Sample code	WA (°)
PU-1.0-Bk	51.5±0.55	PU-2.0-Bk	41.5±0.65	PUR-S0-Bk	72.97 ± 1.23
PU-1.1-BZ	70.2±0.75	PU-2.1-BZ	65.27±0.5	PUR-S1-RZ	62.30 ± 0.73
PU-1.2- o-PhDA	59.35±0.35	PU-2.2-o-PhDA	55.21±0.35	PUR-S2-HQ	57.62 ± 0.82
PU-1.3- di-PhHA	53.89±0.49	PU-2.3-di-PhHA	50.2±0.45	PUR-S3-Py	36.04 ± 0.75

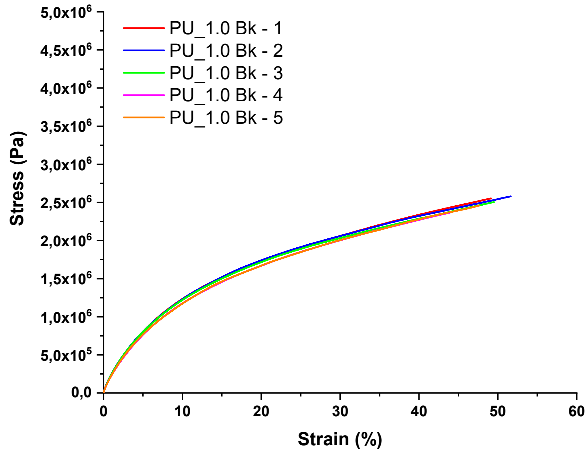

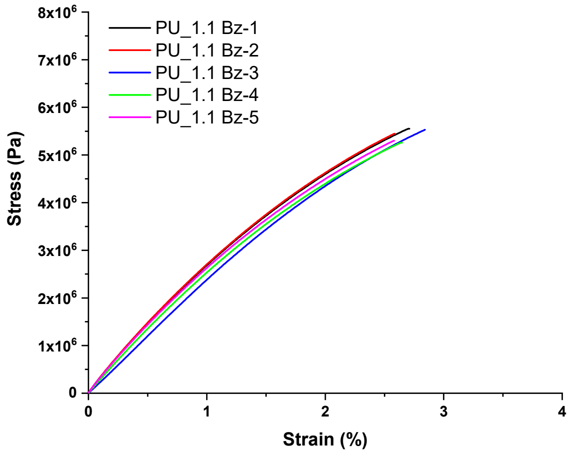

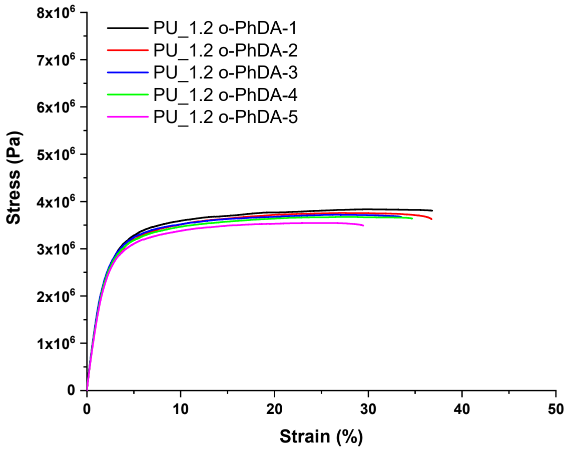

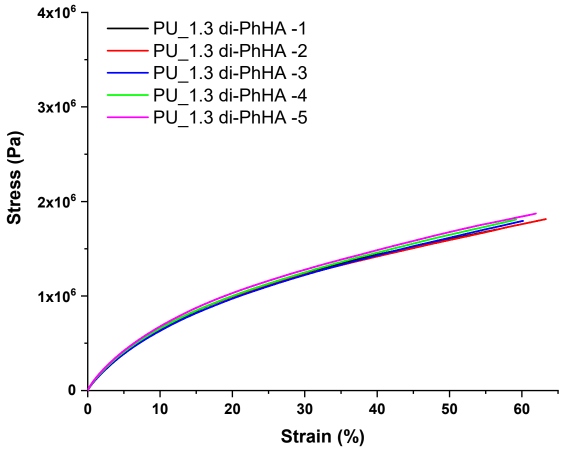

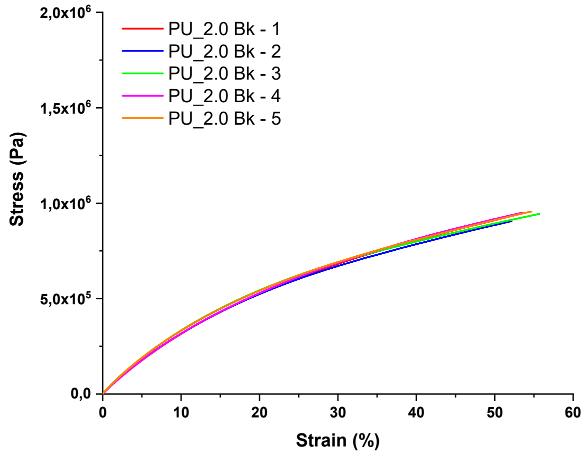

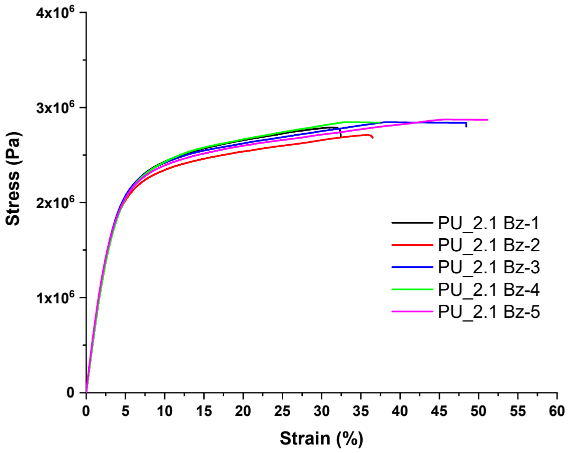

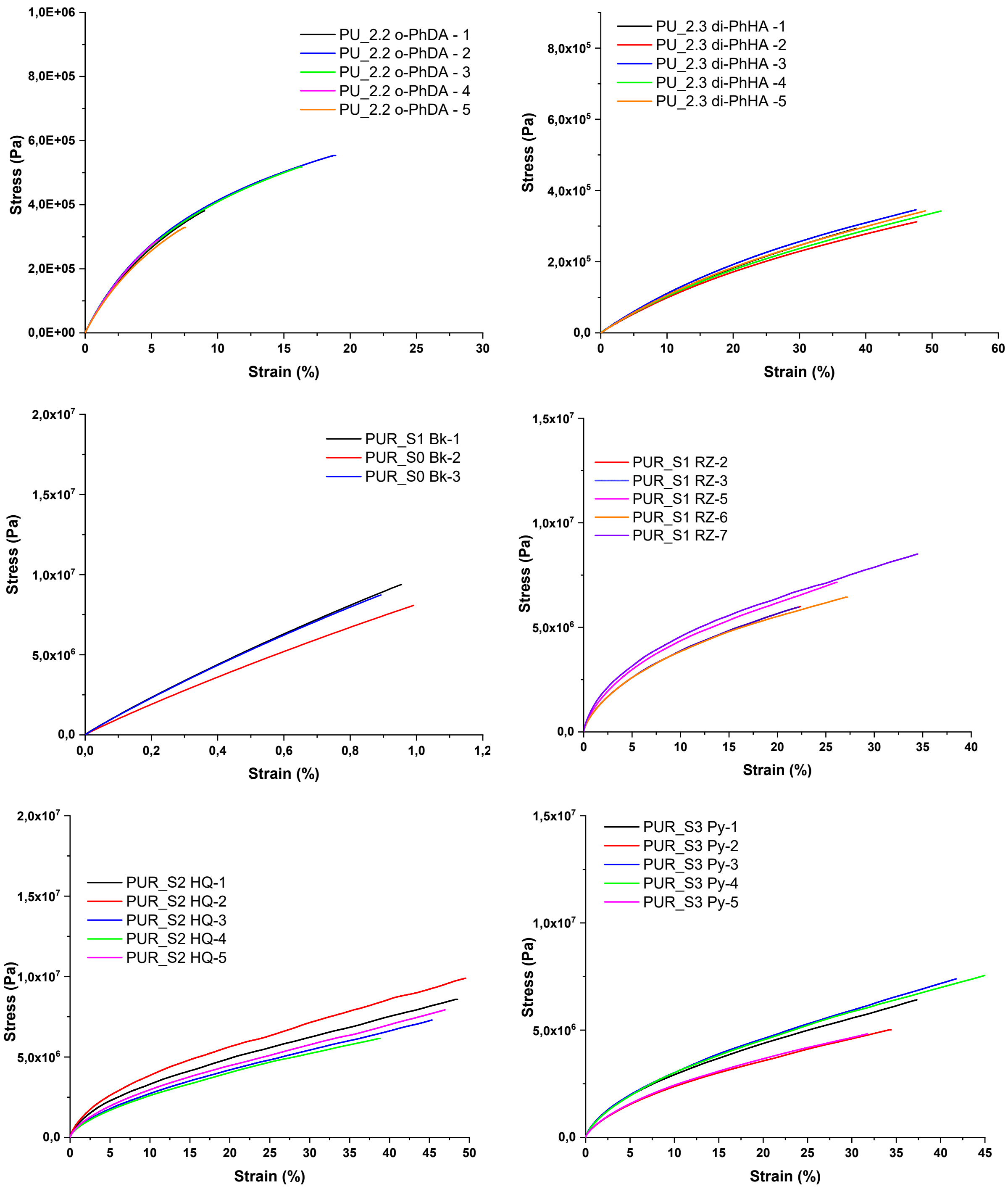

Figure S4. Tensile tests.

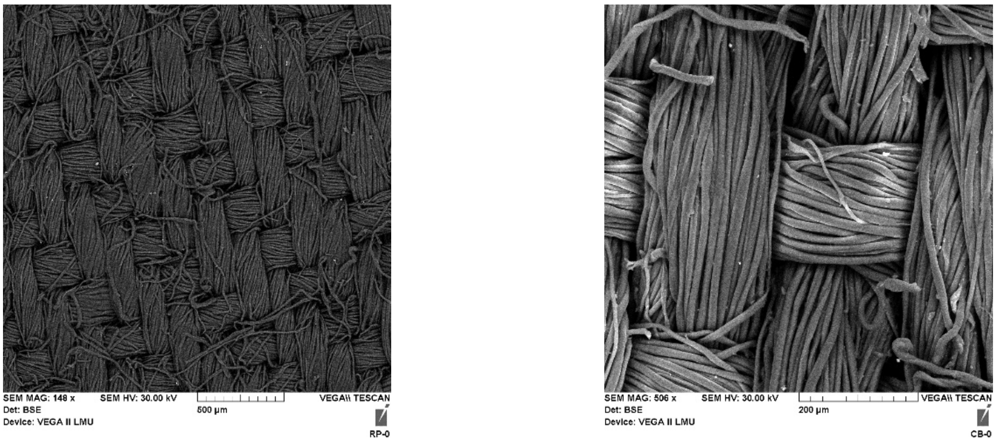

Figure S5. SEM images of uncoated textile specimens.

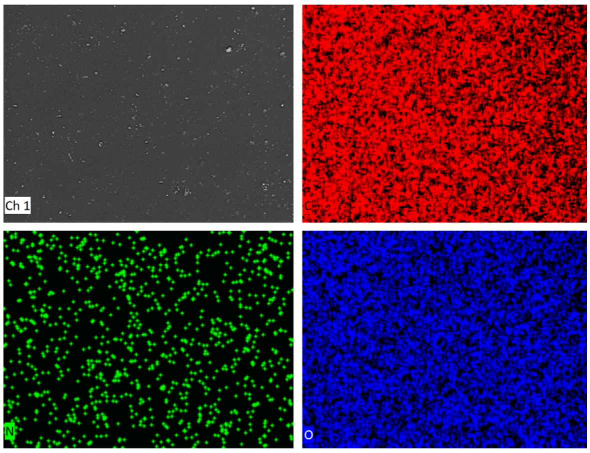

A – neat PU front-view;

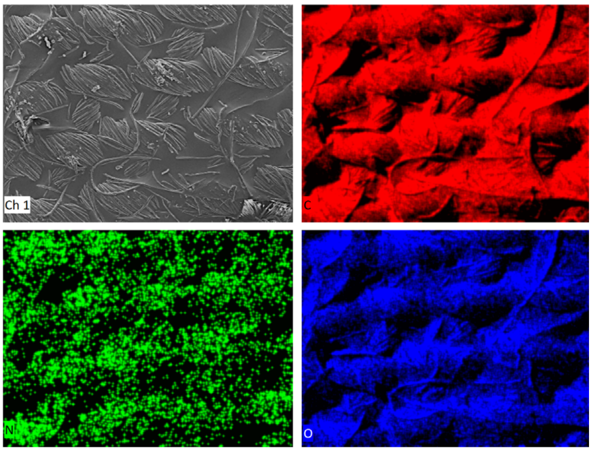

B – textile specimen coated with PU front-view;

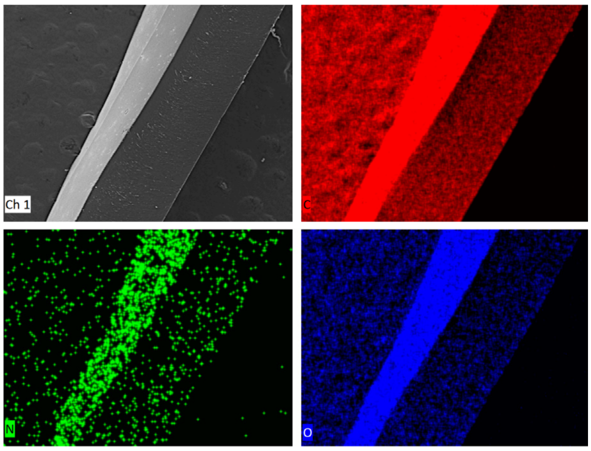

A – neat PU side-view;

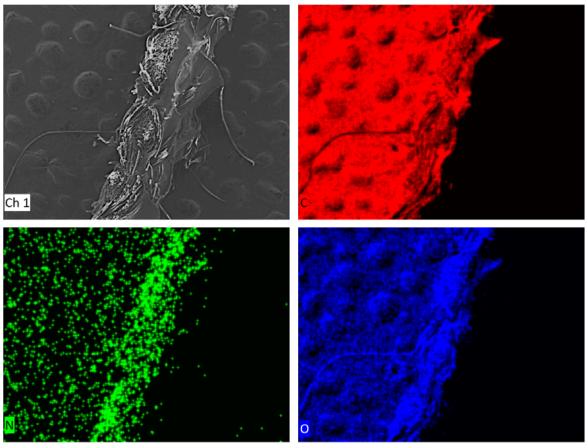

C – textile specimen coated with PU side-view;

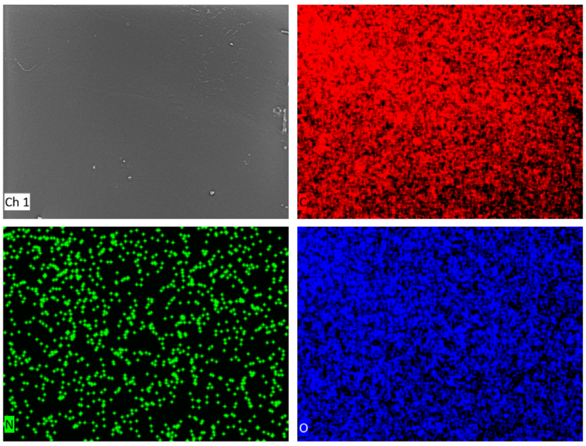

D – neat PUR front-view;

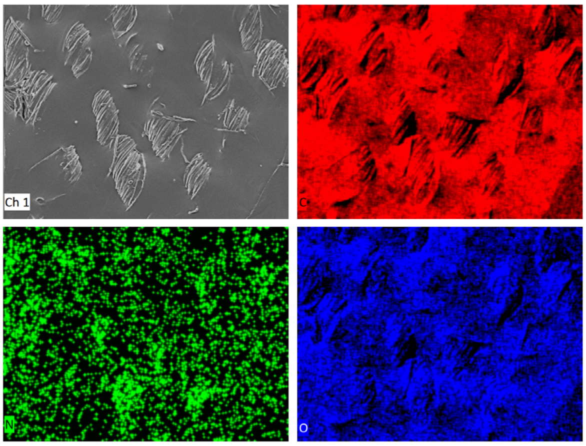

E – textile specimen coated with PUR front-view;

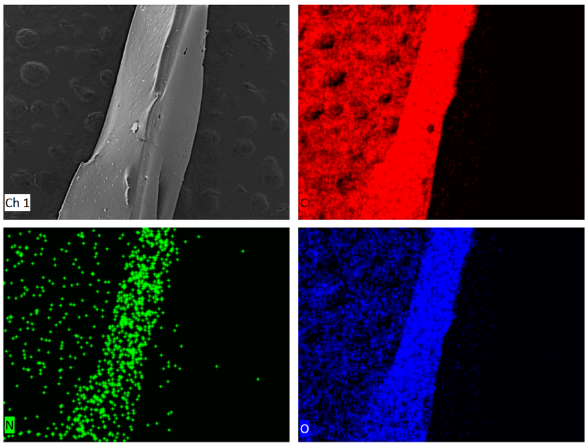

F – neat PUR side-view;

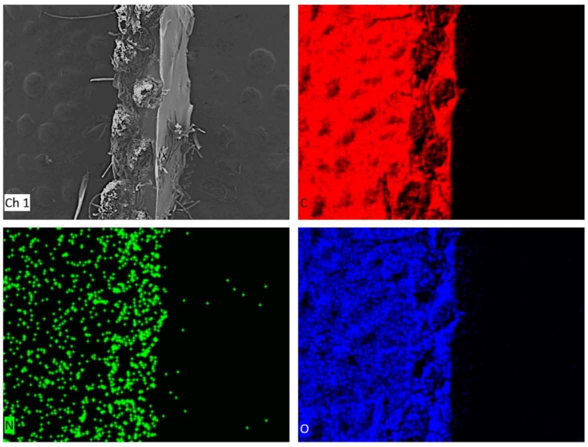

G – textile specimen coated with PUR side-view;

Figure S6. SEM – EDX mapping of the coated textile specimens.

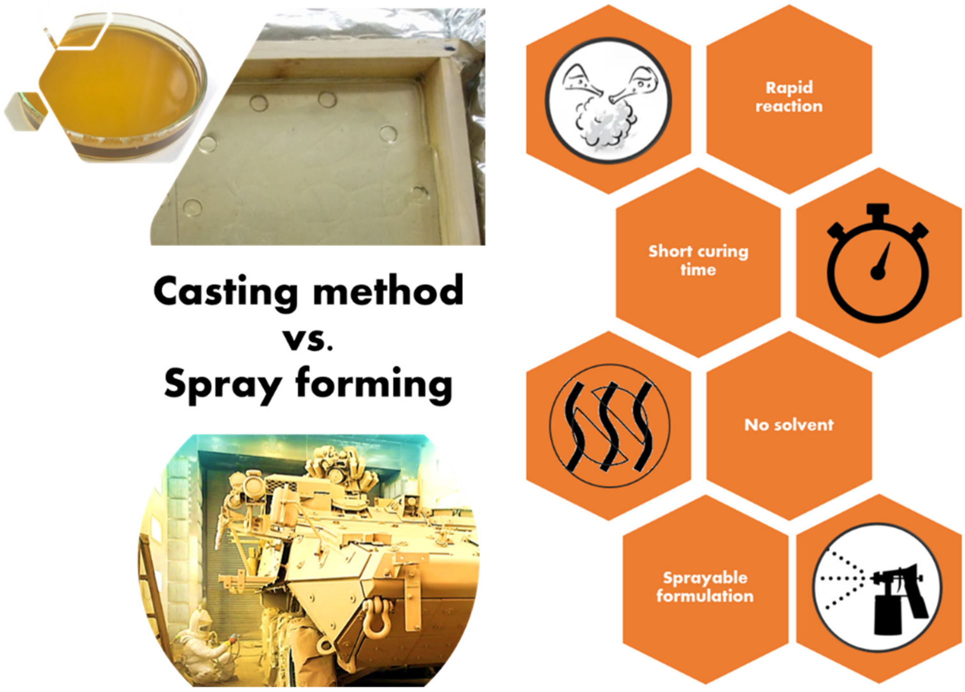

Figure S7. Spray forming advantages for ballistic protection applications.
